# Supplementary material for: Predictive value of three Inflammation-based Glasgow Prognostic Scores for major cardiovascular adverse events in patients with acute myocardial infarction during hospitalization: a retrospective study
Source: PeerJ. 2020 Apr 24;8:e9068. doi: 10.7717/peerj.9068 (PMC7185027; doi:10.7717/peerj.9068)
Supplement: Supplemental Information 2 [file peerj-08-9068-s002.docx]

Predictive value of three Inflammation-based Glasgow Prognostic Scores for major cardiovascular adverse events in patients with acute myocardial infarction during hospitalization

Houyong Zhu, MD, Xiaoqun Xu, MD, Xiaojiang Fang, MD, Zhaodong Li, MD, Tielong Chen, PhD, Jinyu Huang, PhD.

**Supplementary file 2 to the manuscript**

**Contents of supplementary appendix**

[Appendix 1 3](#_Toc1952924396)

[Statistics of missing value and extremesa 3](#_Toc660262824)

[Appendix 2 5](#_Toc989278919)

[ROC analysis of in-hospital MACE for subgroups 5](#_Toc992396559)

[In the STEMI group, three kinds of ROC curves of GPSs predicting MACEs 5](#_Toc1850964511)

[Pairwise comparison of ROC curves in the STEMI group 5](#_Toc712425935)

[In the NSTEMI group, three kinds of ROC curves of GPSs predicting MACEs 7](#_Toc1521357520)

[Pairwise comparison of ROC curves in the NSTEMI group 7](#_Toc1515537458)

[In the HTI-GPS group, three kinds of ROC curves of GPSs predicting MACEs 9](#_Toc334519539)

[Pairwise comparison of ROC curves in the HTI-GPS group 9](#_Toc157704127)

[In the LTI-GPS group, three kinds of ROC curves of GPSs predicting MACEs 11](#_Toc538442091)

[Pairwise comparison of ROC curves in the LTI-GPS group 11](#_Toc100134979)

[In the PPCI group, three kinds of ROC curves of GPSs predicting MACEs 13](#_Toc1488896452)

[Pairwise comparison of ROC curves in the PPCI group 13](#_Toc1403213920)

[In the EPCI group, three kinds of ROC curves of GPSs predicting MACEs 15](#_Toc150942086)

[Pairwise comparison of ROC curves in the EPCI group 15](#_Toc705452295)

[In the non-PCI group, three kinds of ROC curves of GPSs predicting MACEs 17](#_Toc279506978)

[Pairwise comparison of ROC curves in the non-PCI group 17](#_Toc1127043257)

# Appendix 1

## Statistics of missing value and extremesa

|  | N | Mean | Std. Deviation | Missing | | No. of Extremesa | |
| --- | --- | --- | --- | --- | --- | --- | --- |
|  |  |  |  | Count | Percent | Low | High |
| Age | 188 | 68.21 | 14.887 | 0 | 0 | 0 | 0 |
| Hypertension | 188 | 0.72 | 0.451 | 0 | 0 | 0 | 0 |
| Diabetics | 188 | 0.43 | 0.496 | 0 | 0 | 0 | 0 |
| Hemoglobin | 188 | 125.95 | 26.826 | 0 | 0 | 2 | 0 |
| Platelet | 188 | 202.29 | 66.365 | 0 | 0 | 0 | 3 |
| ALT | 188 | 83.82 | 211.12 | 0 | 0 | 0 | 12 |
| CK | 188 | 1223.68 | 1695.864 | 0 | 0 | 0 | 12 |
| CKMB | 188 | 134.91 | 181.0902 | 0 | 0 | 0 | 14 |
| Albumin | 188 | 36.135 | 4.3573 | 0 | 0 | 2 | 0 |
| HS-CRP | 188 | 36.2287 | 55.04936 | 0 | 0 | 0 | 28 |
| LDL | 186 | 2.9675 | 1.02712 | 2 | 1.1 | 0 | 5 |
| Creatinine | 188 | 155.98 | 178.972 | 0 | 0 | 0 | 28 |
| BNP | 162 | 880.703 | 1235.7638 | 26 | 13.8 | 0 | 16 |
| TNI | 188 | 26.948 | 32.46986 | 0 | 0 | 0 | 15 |
| Heart rate | 188 | 80.76 | 17.731 | 0 | 0 | 0 | 6 |
| SBP | 188 | 130.51 | 25.779 | 0 | 0 | 0 | 1 |
| DBP | 188 | 74.16 | 13.059 | 0 | 0 | 2 | 2 |
| EF (Simpson) | 20 | 0.4574 | 0.094459 | 168 | 89.4 | 0 | 0 |
| EF (M) | 90 | 0.58672 | 0.095564 | 98 | 52.1 | 4 | 0 |
| D-dimer | 186 | 1.9945 | 4.82587 | 2 | 1.1 | 0 | 22 |
| Males | 188 |  |  | 0 | 0 |  |  |
| ST segment down | 188 |  |  | 0 | 0 |  |  |
| MACE | 188 |  |  | 0 | 0 |  |  |
| All-cause mortality | 188 |  |  | 0 | 0 |  |  |
| Killip class | 188 |  |  | 0 | 0 |  |  |
| PCI type | 188 |  |  | 0 | 0 |  |  |
| a Number of cases outside the range (Q1 - 1.5*IQR, Q3 + 1.5*IQR). | | | | | | | |

# Appendix 2

## ROC analysis of in-hospital MACE for subgroups

### In the STEMI group, three kinds of ROC curves of GPSs predicting MACEs


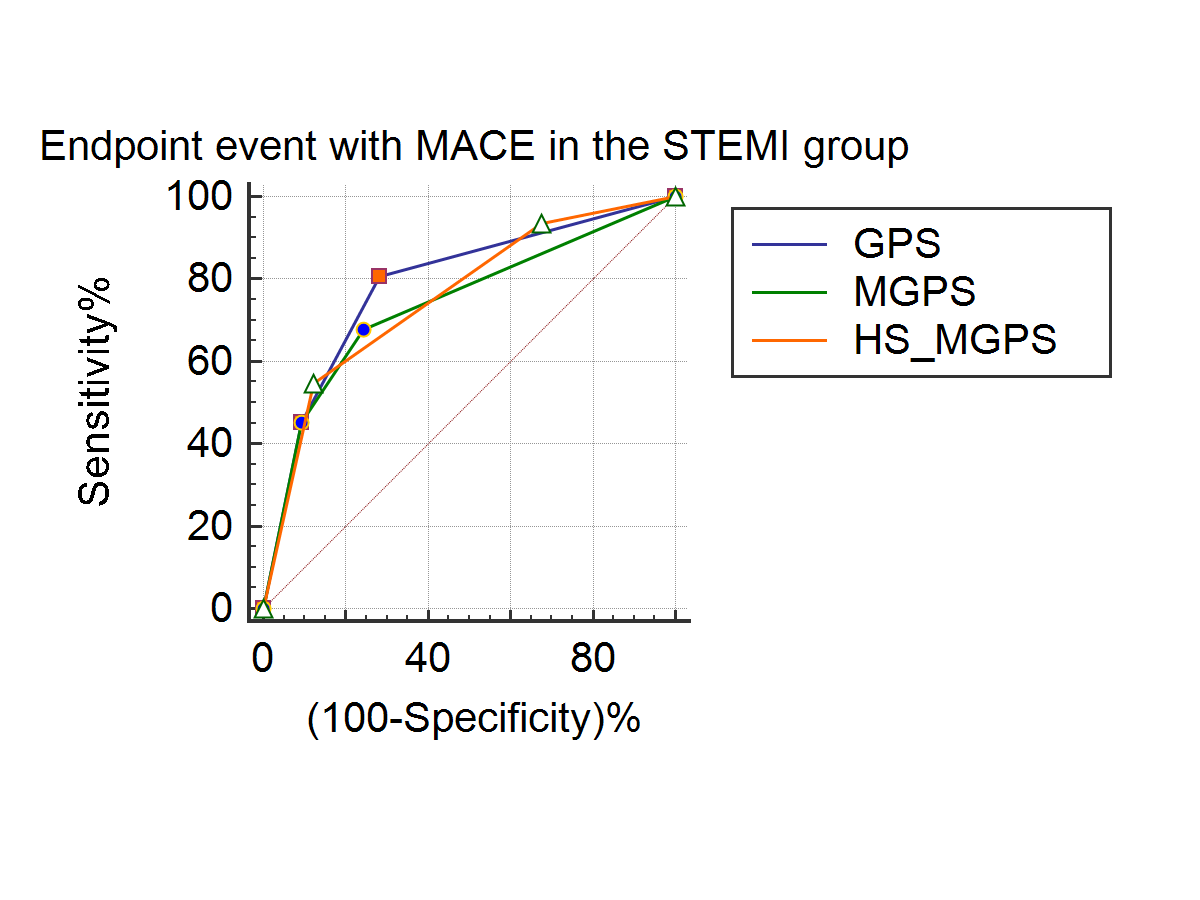


### Pairwise comparison of ROC curves in the STEMI group

| GPS ~ MGPS | |
| --- | --- |
| Difference between areas | 0.0473 |
| Standard Error ^a^ | 0.0279 |
| 95% Confidence Interval | -0.00739 to 0.102 |
| z statistic | 1.695 |
| Significance level | P = 0.0901 |
| GPS ~ HS_MGPS | |
| Difference between areas | 0.0290 |
| Standard Error ^a^ | 0.0376 |
| 95% Confidence Interval | -0.0448 to 0.103 |
| z statistic | 0.770 |
| Significance level | P = 0.4413 |
| MGPS ~ HS_MGPS | |
| Difference between areas | 0.0183 |
| Standard Error ^a^ | 0.0456 |
| 95% Confidence Interval | -0.0710 to 0.108 |
| z statistic | 0.402 |
| Significance level | P = 0.6879 |

^a^ DeLong et al., 1988

### In the NSTEMI group, three kinds of ROC curves of GPSs predicting MACEs


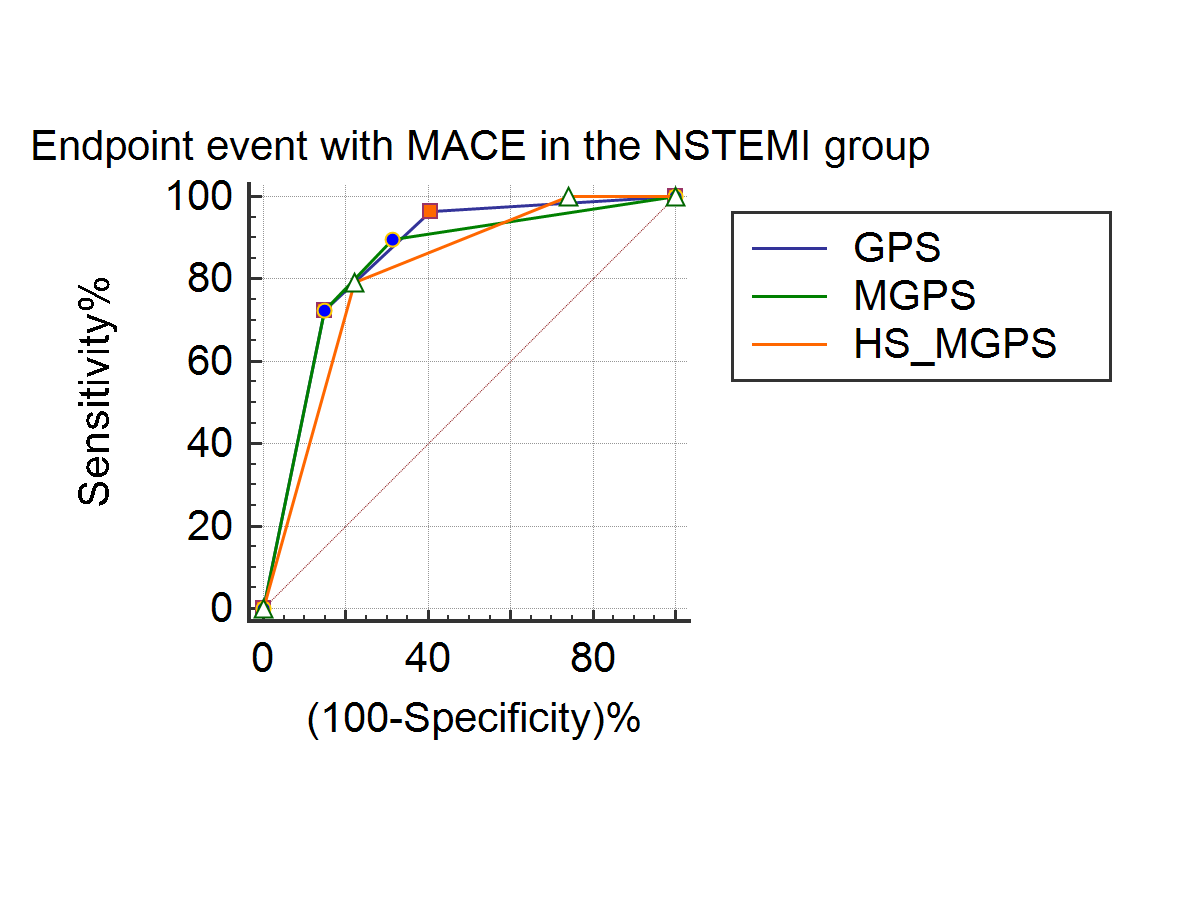


### Pairwise comparison of ROC curves in the NSTEMI group

| GPS ~ MGPS | |
| --- | --- |
| Difference between areas | 0.0166 |
| Standard Error ^a^ | 0.0198 |
| 95% Confidence Interval | -0.0222 to 0.0554 |
| z statistic | 0.839 |
| Significance level | P = 0.4014 |
| GPS ~ HS_MGPS | |
| Difference between areas | 0.0428 |
| Standard Error ^a^ | 0.0259 |
| 95% Confidence Interval | -0.00802 to 0.0936 |
| z statistic | 1.650 |
| Significance level | P = 0.0989 |
| MGPS ~ HS_MGPS | |
| Difference between areas | 0.0262 |
| Standard Error ^a^ | 0.0405 |
| 95% Confidence Interval | -0.0532 to 0.106 |
| z statistic | 0.647 |
| Significance level | P = 0.5178 |

^a^ DeLong et al., 1988

### In the HTI-GPS group, three kinds of ROC curves of GPSs predicting MACEs


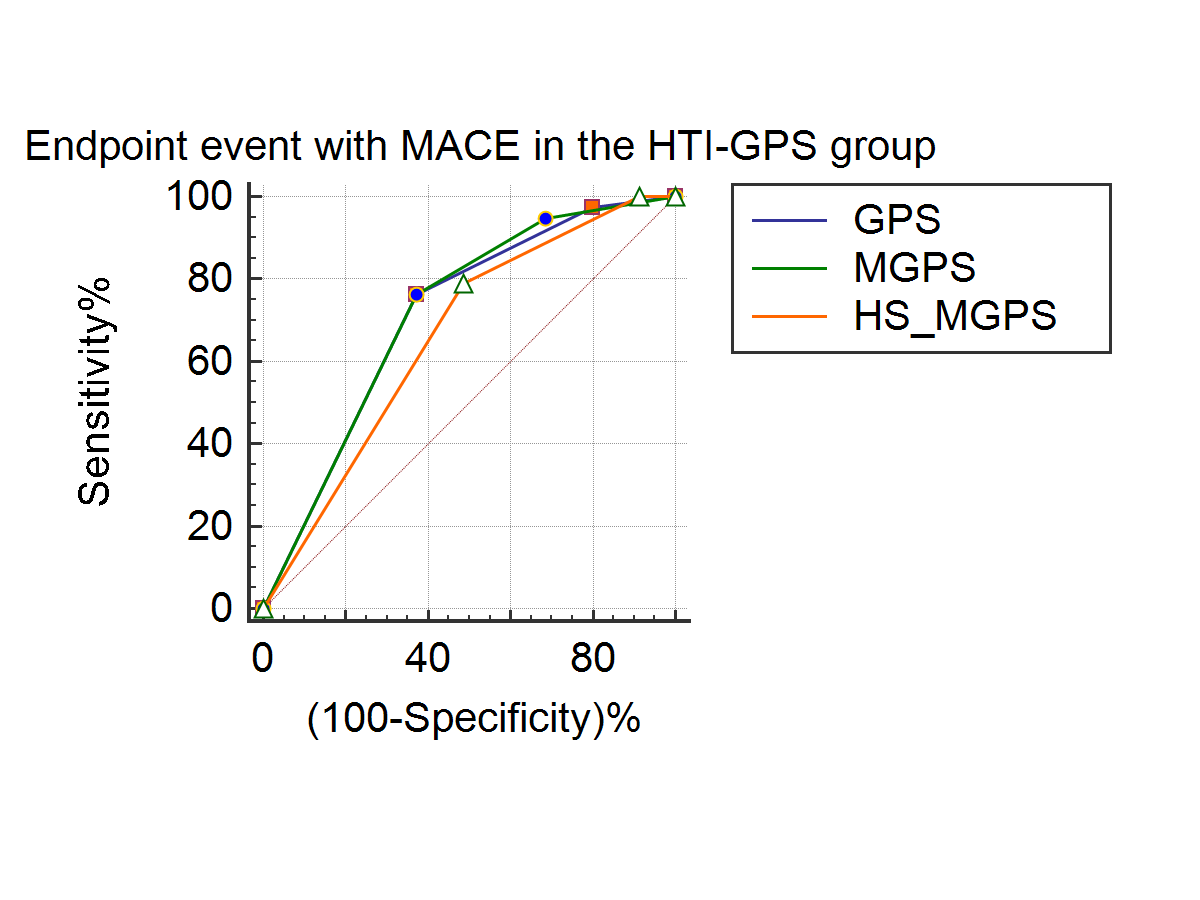


### Pairwise comparison of ROC curves in the HTI-GPS group

| GPS ~ MGPS | |
| --- | --- |
| Difference between areas | 0.00526 |
| Standard Error ^a^ | 0.0102 |
| 95% Confidence Interval | -0.0147 to 0.0252 |
| z statistic | 0.516 |
| Significance level | P = 0.6056 |
| GPS ~ HS_MGPS | |
| Difference between areas | 0.0504 |
| Standard Error ^a^ | 0.0299 |
| 95% Confidence Interval | -0.00818 to 0.109 |
| z statistic | 1.686 |
| Significance level | P = 0.0918 |
| MGPS ~ HS_MGPS | |
| Difference between areas | 0.0556 |
| Standard Error ^a^ | 0.0384 |
| 95% Confidence Interval | -0.0196 to 0.131 |
| z statistic | 1.449 |
| Significance level | P = 0.1474 |

^a^ DeLong et al., 1988

### In the LTI-GPS group, three kinds of ROC curves of GPSs predicting MACEs


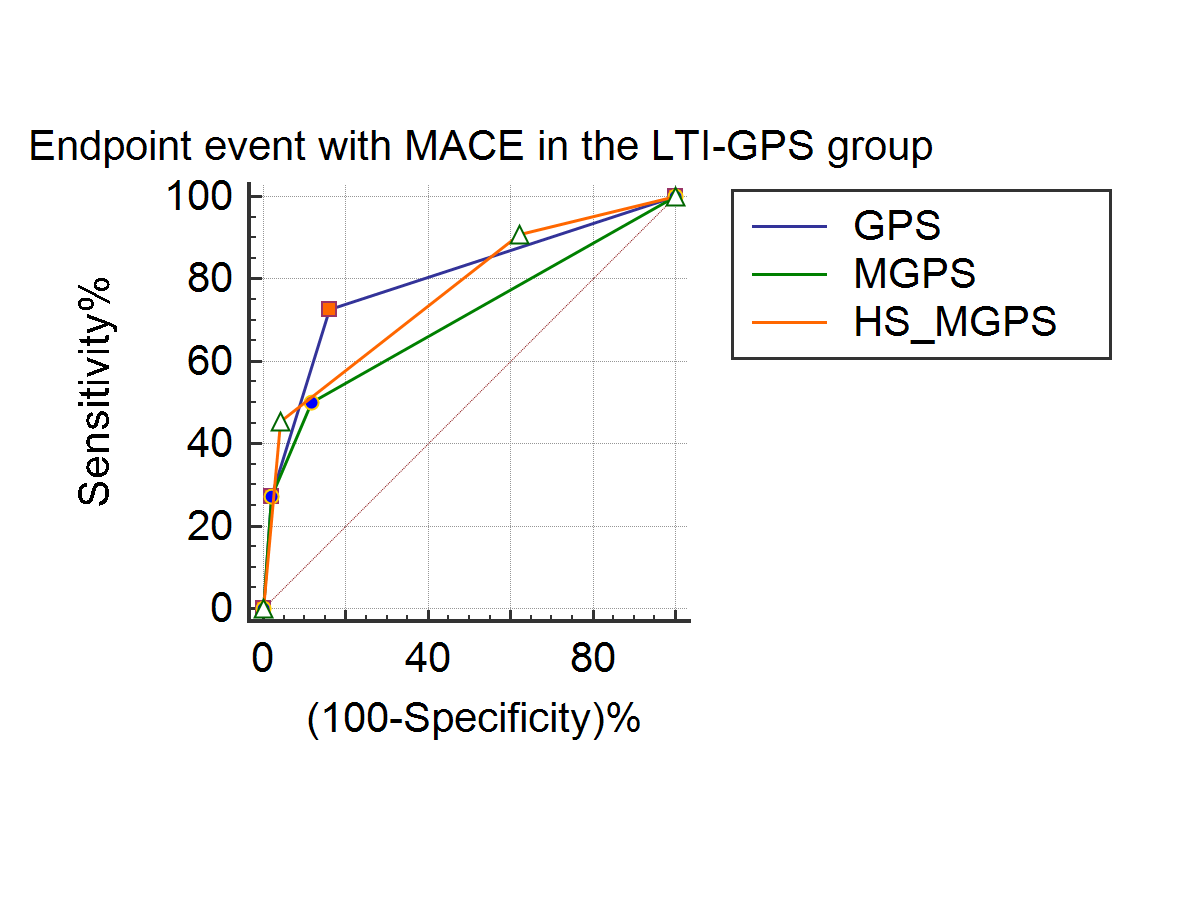


### Pairwise comparison of ROC curves in the LTI-GPS group

| GPS ~ MGPS | |
| --- | --- |
| Difference between areas | 0.0956 |
| Standard Error ^a^ | 0.0448 |
| 95% Confidence Interval | 0.00778 to 0.183 |
| z statistic | 2.134 |
| Significance level | P = 0.0329 |
| GPS ~ HS_MGPS | |
| Difference between areas | 0.0323 |
| Standard Error ^a^ | 0.0532 |
| 95% Confidence Interval | -0.0719 to 0.136 |
| z statistic | 0.607 |
| Significance level | P = 0.5439 |
| MGPS ~ HS_MGPS | |
| Difference between areas | 0.0633 |
| Standard Error ^a^ | 0.0648 |
| 95% Confidence Interval | -0.0638 to 0.190 |
| z statistic | 0.976 |
| Significance level | P = 0.3289 |

^a^ DeLong et al., 1988

### In the PPCI group, three kinds of ROC curves of GPSs predicting MACEs


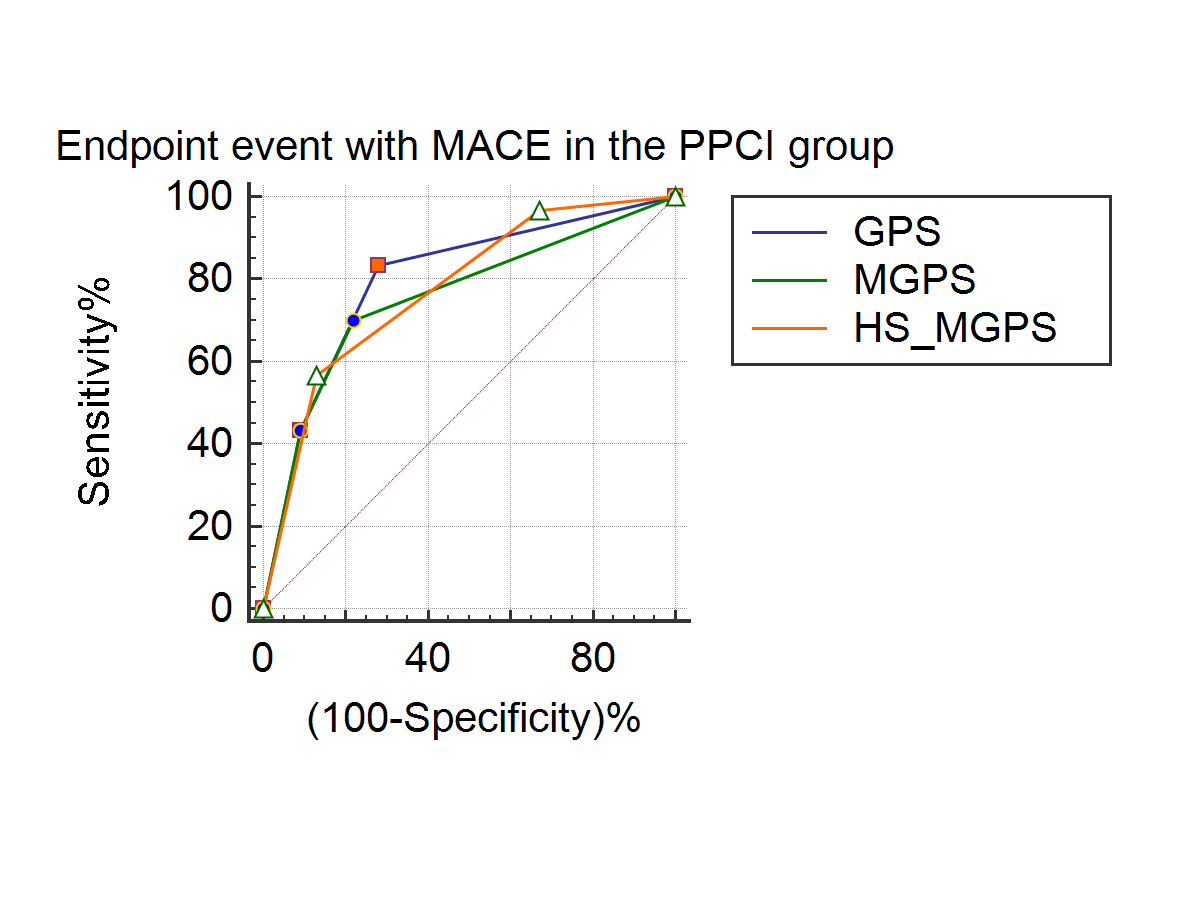


### Pairwise comparison of ROC curves in the PPCI group

| GPS ~ MGPS | |
| --- | --- |
| Difference between areas | 0.0437 |
| Standard Error ^a^ | 0.0287 |
| 95% Confidence Interval | -0.0126 to 0.1000 |
| z statistic | 1.520 |
| Significance level | P = 0.1286 |
| GPS ~ HS_MGPS | |
| Difference between areas | 0.0245 |
| Standard Error ^a^ | 0.0314 |
| 95% Confidence Interval | -0.0371 to 0.0861 |
| z statistic | 0.780 |
| Significance level | P = 0.4357 |
| MGPS ~ HS_MGPS | |
| Difference between areas | 0.0192 |
| Standard Error ^a^ | 0.0494 |
| 95% Confidence Interval | -0.0777 to 0.116 |
| z statistic | 0.388 |
| Significance level | P = 0.6981 |

^a^ DeLong et al., 1988

### In the EPCI group, three kinds of ROC curves of GPSs predicting MACEs


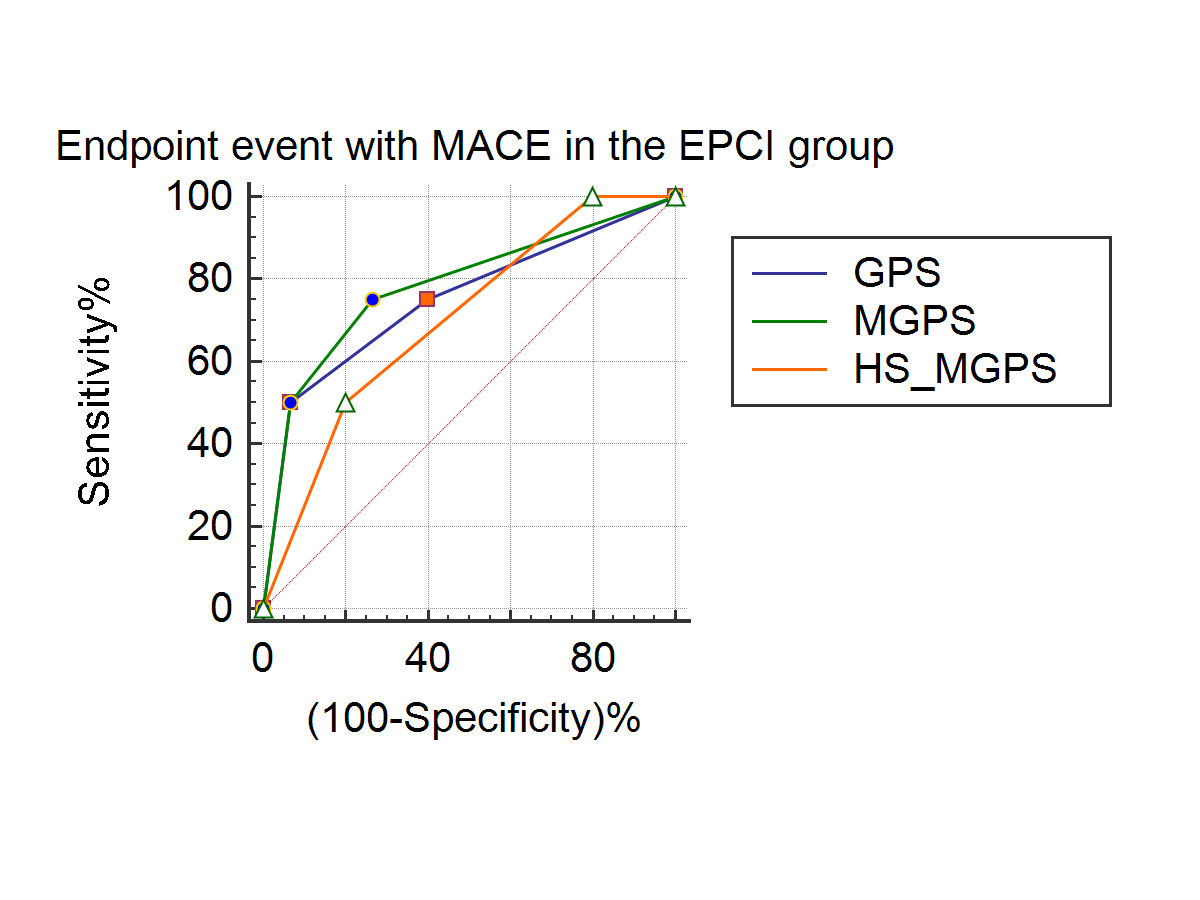


### Pairwise comparison of ROC curves in the EPCI group

| GPS ~ MGPS | |
| --- | --- |
| Difference between areas | 0.0333 |
| Standard Error ^a^ | 0.0298 |
| 95% Confidence Interval | -0.0250 to 0.0917 |
| z statistic | 1.120 |
| Significance level | P = 0.2628 |
| GPS ~ HS_MGPS | |
| Difference between areas | 0.0500 |
| Standard Error ^a^ | 0.106 |
| 95% Confidence Interval | -0.158 to 0.258 |
| z statistic | 0.472 |
| Significance level | P = 0.6369 |
| MGPS ~ HS_MGPS | |
| Difference between areas | 0.0833 |
| Standard Error ^a^ | 0.115 |
| 95% Confidence Interval | -0.143 to 0.309 |
| z statistic | 0.723 |
| Significance level | P = 0.4697 |

^a^ DeLong et al., 1988

### In the non-PCI group, three kinds of ROC curves of GPSs predicting MACEs


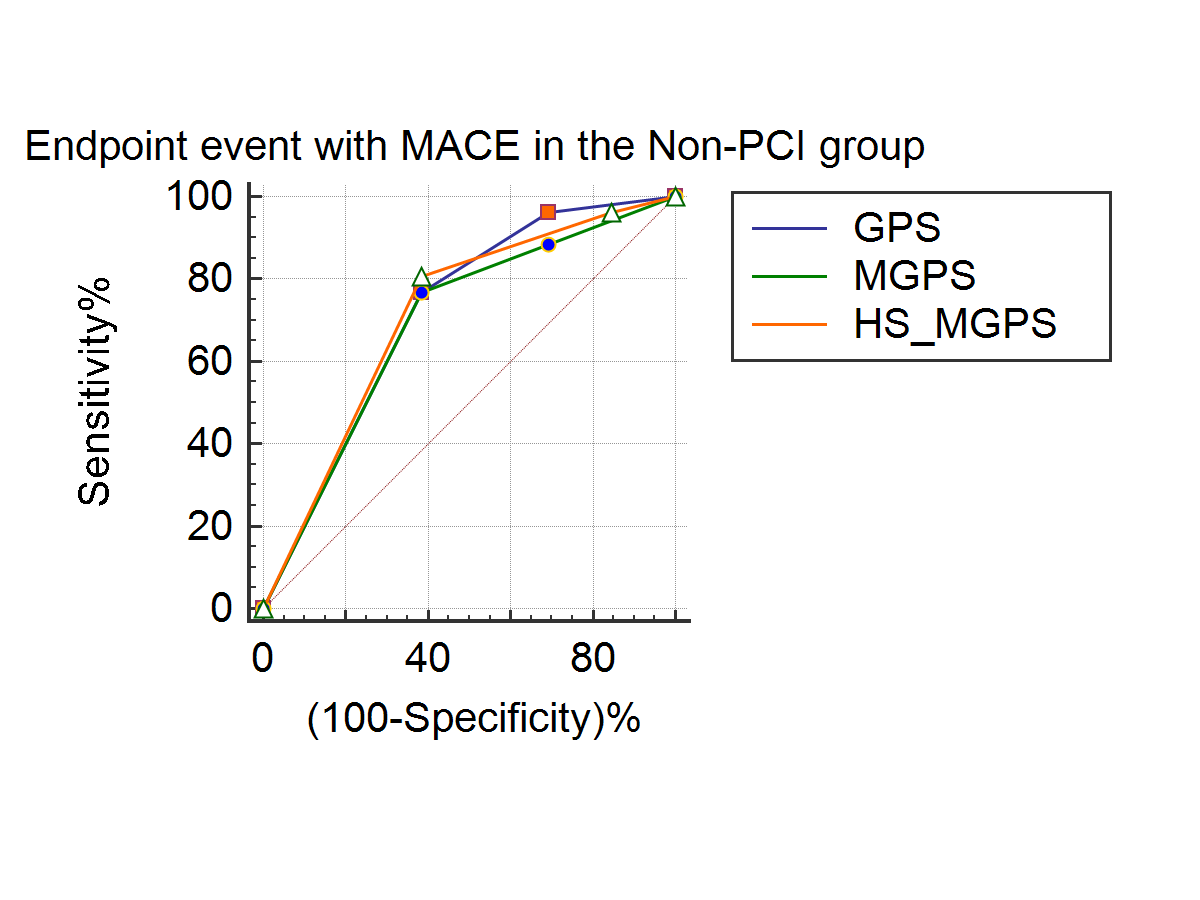


### Pairwise comparison of ROC curves in the non-PCI group

| GPS ~ MGPS | |
| --- | --- |
| Difference between areas | 0.0237 |
| Standard Error ^a^ | 0.0173 |
| 95% Confidence Interval | -0.0102 to 0.0575 |
| z statistic | 1.371 |
| Significance level | P = 0.1704 |
| GPS ~ HS_MGPS | |
| Difference between areas | 0.00148 |
| Standard Error ^a^ | 0.0257 |
| 95% Confidence Interval | -0.0489 to 0.0519 |
| z statistic | 0.0575 |
| Significance level | P = 0.9541 |
| MGPS ~ HS_MGPS | |
| Difference between areas | 0.0222 |
| Standard Error ^a^ | 0.0296 |
| 95% Confidence Interval | -0.0358 to 0.0801 |
| z statistic | 0.750 |
| Significance level | P = 0.4530 |

^a^ DeLong et al., 1988
